# Poly(ADP-ribosyl)ation of Methyl CpG Binding Domain Protein 2 Regulates Chromatin Structure*

**DOI:** 10.1074/jbc.M115.698357

**Published:** 2016-01-15

**Authors:** Annette Becker, Peng Zhang, Lena Allmann, Daniela Meilinger, Bianca Bertulat, Daniel Eck, Maria Hofstaetter, Giody Bartolomei, Michael O. Hottiger, Valérie Schreiber, Heinrich Leonhardt, M. Cristina Cardoso

**Affiliations:** From the ‡ Department of Biology, Technische Universität Darmstadt, 64287 Darmstadt, Germany,; the § Center for Integrated Protein Science at the Department of Biology, Ludwig Maximilians University Munich, 82152 Planegg-Martinsried, Germany,; the ¶ Max Delbrück Center for Molecular Medicine, 13125 Berlin, Germany,; the ‖ Department of Molecular Mechanisms of Disease, University of Zurich, 8057 Zurich, Switzerland, and; ** UMR7242 Biotechnology and Cell Signaling, Laboratory of Excellence Medalis, Strasbourg University, CNRS, Ecole Superieure de Biotechnologie de Strasbourg, BP10413, 67412 Illkirch Cedex, France

**Keywords:** 5-methylcytosine, chromatin remodeling, DNA methylation, heterochromatin, post-translational modification (PTM), MeCP2, PARP-1, chromatin binding and organization, poly(ADP-ribosyl)ation

## Abstract

The epigenetic information encoded in the genomic DNA methylation pattern is translated by methylcytosine binding proteins like MeCP2 into chromatin topology and structure and gene activity states. We have shown previously that the MeCP2 level increases during differentiation and that it causes large-scale chromatin reorganization, which is disturbed by MeCP2 Rett syndrome mutations. Phosphorylation and other posttranslational modifications of MeCP2 have been described recently to modulate its function. Here we show poly(ADP-ribosyl)ation of endogenous MeCP2 in mouse brain tissue. Consequently, we found that MeCP2 induced aggregation of pericentric heterochromatin and that its chromatin accumulation was enhanced in poly(ADP-ribose) polymerase (PARP) 1^−/−^ compared with wild-type cells. We mapped the poly(ADP-ribosyl)ation domains and engineered MeCP2 mutation constructs to further analyze potential effects on DNA binding affinity and large-scale chromatin remodeling. Single or double deletion of the poly(ADP-ribosyl)ated regions and PARP inhibition increased the heterochromatin clustering ability of MeCP2. Increased chromatin clustering may reflect increased binding affinity. In agreement with this hypothesis, we found that PARP-1 deficiency significantly increased the chromatin binding affinity of MeCP2 *in vivo*. These data provide novel mechanistic insights into the regulation of MeCP2-mediated, higher-order chromatin architecture and suggest therapeutic opportunities to manipulate MeCP2 function.

## Introduction

In mammals, methylation of cytosine residues at the dinucleotide CpG is essential for development and is proposed to regulate genome organization and expression. This epigenetic information is recognized and translated by a family of chromatin-organizing proteins containing a conserved methyl CpG binding domain (MBD)^4^ (1, 2). MeCP2, the founding member of the MBD protein family, has been described to function as a transcriptional silencer through association with corepressor complexes mediated by its transcriptional repression domain (TRD) (3–5). Increased expression of MeCP2 in mouse cells induces aggregation of pericentric heterochromatin in a dose-dependent manner, and the clustering ability is mostly dependent on the MBD (6–8). Purified MeCP2 has also been shown to cause compaction of nucleosomal arrays *in vitro* (9).

Mutations within the X chromosome-located *MECP2* gene have been linked to one of the most common human mental retardation disorders in females, Rett syndrome (Online Mendelian Inheritance in Man database no. 321750) (10). Although missense mutations are mostly accumulated within the MBD (amino acids 78–162), the majority of nonsense mutations occur predominantly within the TRD (amino acids 207–310). MeCP2 Rett syndrome-associated mutations have been shown to affect the ability of MeCP2 to bind DNA and to compact polynucleosomal arrays *in vitro* (9, 11) and MeCP2 chromatin binding kinetics *in vivo* (12–14). In addition, we have recently identified MeCP2 mutants with a decreased ability to accumulate at pericentric heterochromatin and/or with decreased heterochromatin clustering potential (14, 15). Despite accumulating evidence in favor of a major role of MeCP2 in controlling large-scale heterochromatin organization, the underlying mechanism and its regulation have so far not been elucidated.

In this study, we found that endogenous MeCP2 from mouse brain tissue is poly(ADP-ribosyl)ated *in vivo*. We identified two distinct MeCP2 domains relevant for poly(ADP-ribosyl)ation and could show that deletion of these modifiable domains increased heterochromatin clustering. Furthermore, we found that PARP-1 deficiency increases the ability of MeCP2 to aggregate and to bind to pericentric heterochromatin. These findings unravel a novel mechanism modulating MeCP2-dependent chromatin organization.

## Experimental Procedures

### 

#### 

##### Expression Plasmids

Mammalian expression constructs coding for GFP- or YFP-tagged full-length rat MeCP2 (MeCP2G) and deletions (MeCP2Y.3, MeCP2G.8, and MeCP2G.9) have been described previously (6, 16). Additional mammalian expression constructs were generated in the following way. Deletion constructs MeCP2G.11–15 designed with flanking XhoI and BamHI sites according to the sequence of MeCP2G were custom-synthesized into pPCR Script (Sloning Bio Technology, Puchheim, Germany) and subcloned into the XhoI and BamHI sites of MeCP2G.6 (8). MeCP2G.16–18 were generated using site-directed mutagenesis as described previously in detail (17, 18). For expression in Sf9 insect cells, the Bac-to-Bac baculovirus expression system (Invitrogen) was used, employing a rat MeCP2G construct and a GFP construct described previously (16, 19).

To express PARP-1 with an N-terminal strep tag, a sequence encoding the Strep-tactin target peptide Strep Tag III (20) was synthesized into pPCR-Script-Amp (Entelechon, Bad Abbach, Germany) flanked by BamHI and NotI sites and subcloned into pFastBac1 using the same sites. Human full-length PARP-1 and deletion constructs were generated by PCR amplification using primers with NotI and XhoI sites and subcloned in-frame with the strep tag in the pFastBac1 vector.

##### Cell Culture and Transfection

Pmi28 diploid mouse myoblasts were cultured as described before (21). Cells were grown to 70–80% confluency on 16-mm glass coverslips in 6-well plates and transfected using TransFectin (Bio-Rad). For transfection, 3 μg of plasmid DNA together with 3 μl of TransFectin were incubated in serum-free medium for 20 min at room temperature and added to the cells. After incubation at 37 °C for 4 h, the medium was changed, and the culture was incubated at 37 °C overnight. For PARP inhibition assays, cells were treated with 10 mm 3AB (Alexis Biochemicals, Lörrach, Germany) immediately after medium change for 12–15 h. Within this time, medium plus inhibitors were refreshed every 3 h. Transfected cells were fixed with 3.7% formaldehyde in 1× PBS for 10 min. In the case of PARP inhibition, 10 mm 3AB was also added to the solutions during fixation. All washing steps after fixation were performed with 1× PBS plus 0.01% Tween 20. Cells were counterstained with DAPI, followed by mounting in Vectashield (Vector Laboratories, Burlingame, CA). HEK 293-EBNA (Epstein-Barr virus nuclear antigen-1) cells (Invitrogen) were cultured and transfected as described previously (8).

Wild-type and PARP-1^−/−^ mouse embryonic fibroblast (MEF) cells (22) were cultured in Dulbecco's modified Eagle's medium (DMEM; 1 g/liter glucose) supplemented with 10% fetal bovine serum, transfected with TransFectin (Bio-Rad; Hercules, CA, USA) or poly-ethylenimine (PEI, 1 mg/ml in ddH2O, neutralized with HCl; Sigma-Aldrich, St. Louis, MO, USA) (15) and fixed with formaldehyde as above.

For fluorescence recovery after photobleaching (FRAP) and *in situ* extraction experiments, wild-type and PARP-1^−/−^ MEF cells were transfected by electroporation. Briefly, the cell pellet was resuspended in 100 μl of Amaxa transfection buffer (50 mm KCl, 15 mm MgCl_2_, 120 mm Na_2_HPO_4_ and 50 mm mannitol) with 2 μg of plasmid DNA. The mixture was then transferred to an Amaxa cuvette and transfected in an Amaxa Nucleofector® using the B-32 program for wild-type cells and the B-16 program for PARP-1^−/−^ cells. Following transfection, the cells were immediately transferred into a μ-Dish^35 mm^ (ibidi GmbH, Munich, Germany) with 3 ml of prewarmed and pre-equilibrated DMEM and incubated for 20 h.

Sf9 insect cells (Invitrogen) were maintained in EX-CELL 420 insect serum free medium (SAFC, Hampshire, UK) supplemented with 10% fetal bovine serum with shaking at 100 rpm and at 28 °C. Transfection of Sf9 cells to produce a recombinant baculovirus was performed using Cellfectin (Invitrogen) according to the instructions of the manufacturer.

##### Microscopy and Image Analysis

For chromocenter counting, fixed cells were examined on a Zeiss Axiovert 200 epifluorescence microscope. Image stacks (0.5-μm Z interval) were acquired with a ×63 Plan-Apochromatic numerical aperture (NA) 1.4 or ×40 Plan-Neofluar NA 1.3 oil immersion phase-contrast objectives and a PCO Sensicam QE cooled charge-coupled device camera. Images were processed with Adobe Photoshop and ImageJ (http://imagej.nih.gov/ij/). Three-dimensional rendering of image stacks was performed using AMIRA (Visage Imaging Inc., San Diego, CA) software. Image stacks were analyzed for chromocenter numbers as described in detail before (14).

To evaluate heterochromatin accumulation ability, confocal Z stacks were acquired using an UltraView VoX spinning disc system (PerkinElmer Life Sciences) on a Nikon Ti microscope equipped with an oil immersion ×60 Plan-Apochromat NA 1.45 objective lens (Nikon, Tokyo, Japan) (voxel size, 0.12 × 0.12 × 0.5 μm) and a 14-bit electron multiplying cooled charge-coupled device camera (catalog no. C9100-50, Hamamatsu Photonics K.K., Hamamatsu City, Japan). Z stacks were analyzed using Volocity 5.5 software (PerkinElmer Life Sciences). The chromocenter and nucleoplasm were segmented by intensity-based thresholding (Fig. 3). Accumulation at chromocenters was calculated from the ratio of the mean gray value at chromocenters to the mean gray value in the nucleoplasm. Accumulation values from both wild-type and PARP-1^−/−^ cells were then normalized to the median accumulation in wild-type cells.

To evaluate the binding kinetics of fluorescently tagged MeCP2 and deletion mutants in wild-type and PARP-1^−/−^ cells, a whole chromocenter was photobleached using an UltraVIEW VoX spinning disc system (PerkinElmer Life Sciences) mounted on a Nikon Ti microscope equipped with an oil immersion ×60 Plan-Apochromat NA 1.45 objective lens as described before (23). Quantitative evaluation was performed using ImageJ, and fluorescence intensity normalization and curve fitting were performed with easyFRAP software as described before (23). T half-values were extracted from the single exponential fitting, and plots were generated with RStudio.

To evaluate the extractability of fluorescently tagged MeCP2 and deletion mutants in wild-type and PARP-1^−/−^ cells, *in situ* extractions were performed, and release of MeCP2 was measured in real time. The assay was performed as described before with the following exceptions (14). Live-cell imaging was performed on an UltraVIEW VoX spinning disc system (PerkinElmer Life Sciences) mounted on a Nikon Ti microscope equipped with an oil immersion ×60 Plan-Apochromat NA 1.45 objective lens. The cells were washed once with PBS/EDTA and imaged. Then the solution was changed to PBS containing 0.5% Triton X-100. Confocal Z stacks were acquired at 2-min time intervals for MeCP2G and MeCP2Y.1 for 14 min and 40-s intervals for MeCP2Y.3 for 2 min. Quantifications were performed using Volocity (PerkinElmer Life Sciences). The total fluorescence intensity signal at the chromocenters was calculated for each time point, and, for each cell, the fluorescence intensity was normalized to the total intensity of chromocenter before Triton X-100 treatment.

##### In Vivo Binding Assays

HEK 293-EBNA (Invitrogen) or MEF cells (22), transfected with expression plasmids as indicated, were pelleted after washing with 1× PBS, and lysis was performed for 10 min on ice. To disrupt protein-DNA associations and, thus, extract higher protein amounts, buffer B (25 mm Tris-HCl (pH 8.0), 1 m NaCl, 50 mm glucose, 10 mm EDTA, 0.2% Tween 20, and 0.2% Nonidet P-40) was used and supplemented with protease inhibitors (Complete Mini, Roche).

Mouse whole brain tissue (3 months old, catalog no. C57BL/6N, Charles River Laboratories, Inc., Wilmington, MA) was first fractionated to obtain pure nuclei. Tissue (6 g) was first homogenized in a 0.25 m sucrose solution (20 mm triethanolamine-HCl (pH 7.6), 30 mm KCl, 5 mm MgCl_2_, 0.1 mm PMSF, and 1 mm DTT). After centrifugation at 1000 × *g* for 10 min, the pellet was resuspended in 2.1 m sucrose solution followed by centrifugation at 50,000 × *g* for 40 min. The pellet was again dissolved in 0.26 m sucrose solution, and centrifugation was done at 1000 × *g* for 10 min. The isolated nuclei were incubated in buffer B for 15 min on ice. 500 μl of the extract was diluted 1:4 with buffer C (25 mm Tris-HCl (pH 8.0), 50 mm glucose, 10 mm EDTA, 0.2% Tween 20, and 0.2% Nonidet P-40) to obtain an NaCl concentration of 250 mm. After centrifugation (20,000 × *g*, 15 min, 4 °C), rabbit polyclonal anti-MeCP2 antibody (40 μg) (16) or chromatographically purified rabbit IgG (40 μg, Organon Teknika Corp., catalog no. 55944, Durham, NC) was added to the supernatant and incubated for 1.5 h while rotating at 4 °C. To pull down the immunocomplexes, 50 μl of protein A-agarose beads (Fast Flow, Upstate, Temecula, CA), equilibrated with the corresponding buffer, was added and incubated for 1 h.

For purification of MeCP2 from mouse brain tissue employing Tris-NTA-coupled beads (a gift from R. Tampé, Goethe University, Frankfurt, Germany), ∼1 × 10^7^ mouse brain nuclei in 1× PBS were subjected to centrifugation (14,000 rpm, 10 min, 4 °C). The nuclear pellet was dissolved in 0.2% Triton X-100 in 1× PBS supplemented with protease inhibitors as described in Ref. 19, incubated for 10 min on ice and afterwards washed three times with 1× PBS. 300 μl of binding buffer (20 mm imidazole and 0.5 m NaCl in 1× PBS) was added, and sonification was performed twice for 20 s at 70% intensity followed by centrifugation (14,000 rpm, 10 min, 4 °C). 120 μl of Tris-NTA-coupled beads (NHS-activated Sepharose 4 Fast Flow, GE Healthcare) was washed three times with binding buffer and activated for 15–20 min with NiSO_4_-hexahydrate (20 mm), added to the protein lysate, and incubated overnight at 4 °C under rotation. To elute the proteins from the beads, the beads were washed with 40 mm imidazole for 15 min.

To purify endogenous MeCP2 from mouse brain tissue using boronic acid beads, mouse brain tissue was lysed in hot lysis buffer (5 mm Tris HCl (pH 8), 250 mm NaCl, 1% SDS, 0.1% Nonidet P-40, 5 mm MgCl_2_, and 0.5 mm EDTA) supplemented with protease inhibitors (Roche), the poly(ADP-ribose) glycohydrolase inhibitor RBPI-4 (provided by Paul J. Hergenrother (24)), and 100 μm 3AB (Alexis Biochemicals), followed by centrifugation (14,000 rpm, 10 min, room temperature). 30 μl of boronic acid bead slurry (Chemicell) was washed twice in lysis buffer, added to each lysate, and incubated for 30 min at room temperature, followed by three washes in lysis buffer and one wash in water before proceeding with SDS-PAGE and Western blotting.

For immunoprecipitation using the GFP binder (ChromoTek, Planegg-Martinsried, Germany, Ref. 25), 50 μl of protein A-agarose beads was incubated with 100 μg of GFP binder for 1 h, added to the extract, and again incubated for 1 h at 4 °C while rotating. After a short spin, the supernatant was removed, and the beads were washed three times with 500 μl of the same buffer used during cell lysis. The beads were resuspended in 1× SDS-containing sample buffer, boiled for 10 min at 95 °C, and analyzed by SDS-PAGE electrophoresis followed by Western blotting.

##### Purification of Proteins

Sf9 insect cells (Invitrogen) were infected with recombinant baculovirus (P3 stock) and incubated at 28 °C with shaking for 5 days. The cells were pelleted by centrifugation (200 × *g*, 5 min, 4 °C) and resuspended in either buffer B or buffer D (PBS containing 300 mm NaCl and 0.05% Nonidet P-40). All buffers were supplemented with protease inhibitors (Complete Mini, Roche). After incubation on ice for 10 min, cells were disrupted with a high-pressure homogenizer (EmulsiFlex-C5, Avestin, Ottawa, Ontario, Canada), followed by centrifugation at 14,000 × *g* for 30 min.

Strep-tagged recombinant proteins were purified by incubating the supernatant with 500 μl of Strep-Tactin-Sepharose beads (IBA, Göttingen, Germany) for 3 h at 4 °C on a rotary shaker. To elute strep-tagged proteins, the beads were incubated with D-Desthiobiotin (0.5 mg/ml, IBA) dissolved in 1× PBS for 30 min at 4 °C. After centrifugation (200 × *g*, 2 min), beads were separated from the eluate containing the purified proteins.

GFP fusion proteins were immobilized using the GFP-Trap (ChromoTek) as described previously (25).

##### Western Blotting Analysis

Western blotting analysis was performed as described previously (26) using a PVDF membrane (Bio-Rad). Immunoreactive bands were visualized using either an ECL Plus or ECL Advanced Western blotting detection kit (GE Healthcare). The following primary antibodies were used for Western blotting analysis: rabbit polyclonal anti-MeCP2 (Upstate, catalog no. 07-013), mouse monoclonal anti-GFP (Roche, catalog no. 11814460001), mouse monoclonal anti-PARP-1 (F-2, catalog no. sc-8007, Santa Cruz Biotechnology), and mouse monoclonal anti-PAR (Trevigen, Gaithersburg, MD, catalog no. 4335-MC). Secondary antibodies used were horseradish peroxidase-conjugated anti-mouse IgG (GE Healthcare, catalog no. NA 931) and horseradish peroxidase-conjugated anti-rabbit IgG (Sigma, catalog no. A-0545).

##### In Vitro Ribosylation Assay

*In vitro* ribosylation analysis of recombinant GFP, MeCP2G, or GFP-tagged MeCP2 deletions immobilized onto GFP-Trap beads (ChromoTek) were performed as described in Ref. 27 with the following modifications. Purified st-PARP-1 (50 ng) from Sf9 cells, 20 μm cold NAD^+^ in addition to [α-^32^P]NAD^+^, and 100 ng of DNase I-activated DNA (Alexis Biochemicals) were used. After the reaction, the proteins were washed three times with buffer B to disrupt binding to st-PARP-1.

##### Quantification of Overexpressed MeCP2G in Mouse Cells

Mouse myoblast cells were transfected with plasmids coding for GFP-tagged MeCP2 using FuGENE HD transfection reagent (Promega; Madison, WI) according to the instructions of the manufacturer. Defined amounts of MeCP2G-expressing cells were separated from untransfected cells and counted using flow cytometry.

Recombinant MeCP2G, extracted from insect cells using buffer B, was immobilized to GFP-Trap beads (ChromoTek). To determine the concentration of immobilized MeCP2G to obtain an MeCP2G standard, SDS-PAGE was performed, followed by staining of the protein using Coomassie Brilliant Blue. In parallel, defined amounts of a BSA standard were analyzed by SDS-PAGE with subsequent Coomassie Brilliant Blue staining. Mean intensity value of BSA standard per ng was calculated and used to determine the amount of MeCP2G.

To calculate the amount of MeCP2G overexpressed in mouse myoblast cells, defined amounts of recombinant MeCP2G standard and MeCP2G extracted from defined numbers of sorted mouse myoblasts were analyzed by SDS-PAGE and quantitative Western blotting with mouse anti-GFP (Roche) and anti-mouse IgG-Cy5 (Jackson ImmunoResearch Laboratories) antibodies using fluorescence imaging (Storm 860, Molecular Dynamics).

## Results

### 

#### 

##### Endogenous MeCP2 from Mouse Brain Tissue Is Poly(ADP-ribosyl)ated in Vivo

Over the last years, several posttranslational modifications have been described for MeCP2. Among them, phosphorylation of MeCP2 has been implicated to affect MeCP2 chromatin binding and neurological functions (28–31).

**FIGURE 1.**
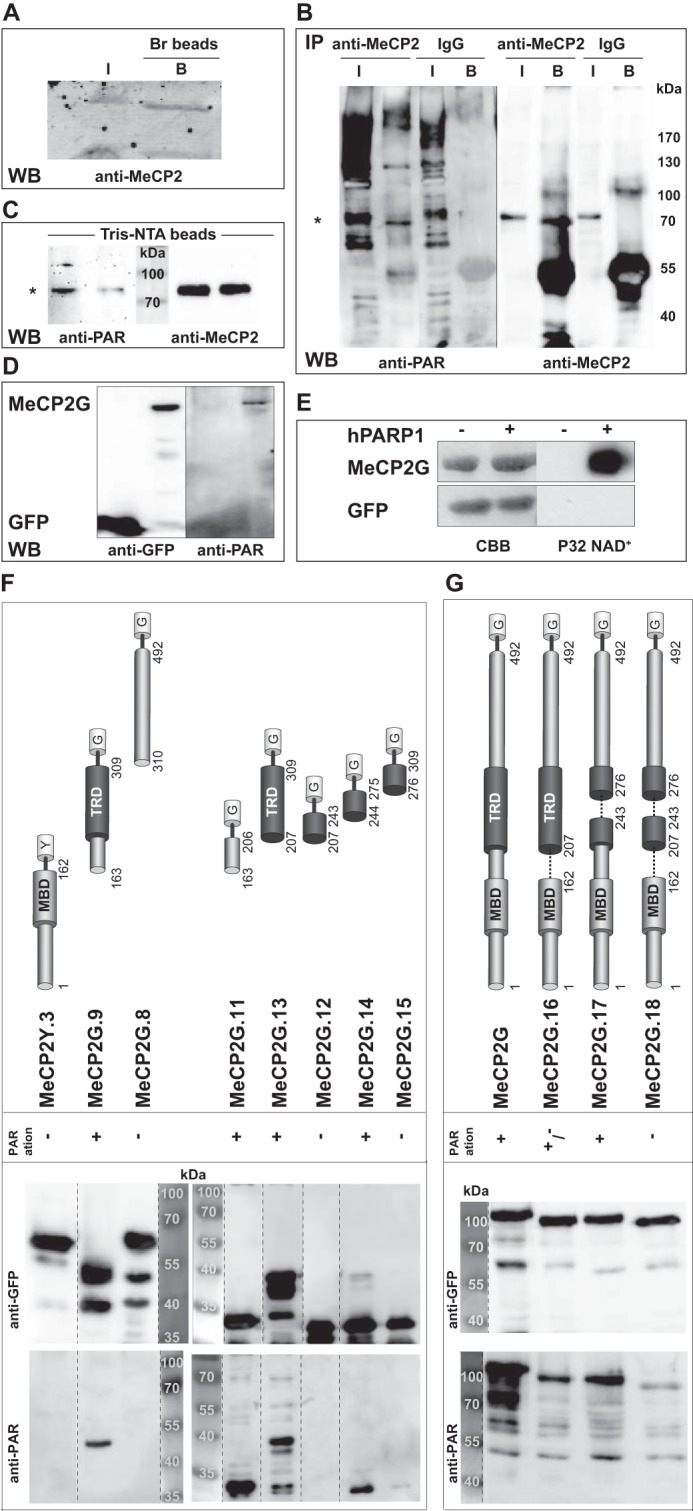
**Endogenous MeCP2 is poly(ADP-ribosyl)ated in the mouse brain.** *A*, endogenous MeCP2 from mouse brain extracts was enriched using boronic acid beads (*Br*) and input (*I*) and bound (*B*) fractions analyzed by Western blotting (*WB*) with anti MeCP2 antibody. *B*, immunoprecipitations (*IP*) of WT mouse brain extracts were performed with the indicated antibodies and analyzed for poly(ADP-ribosyl)ation of endogenous MeCP2 by Western blotting with anti-PAR followed by anti-MeCP2 antibodies. The *asterisk* indicates the expected size of endogenous MeCP2. *C*, endogenous MeCP2 from mouse brain extracts was enriched through Tris-NTA-coupled beads and analyzed by Western blotting using anti-PAR followed by anti-MeCP2 antibodies. The *asterisk* indicates the expected size of endogenous MeCP2. *D*, GFP and MeCP2-GFP were expressed in HEK293-EBNA cells. After immunoprecipitation with GFP-Trap, poly(ADP-ribosyl)ation of the precipitated proteins was checked via Western blotting with anti-poly(ADP-ribose) (*anti-PAR*) followed by anti-GFP antibodies. *E*, recombinant immobilized GFP and MeCP2-GFP proteins were incubated with [α-^32^P]NAD^+^ and DNase I-treated calf thymus DNA with or without purified st-hPARP-1. After SDS-PAGE, poly(ADP-ribosyl)ation was detected by autoradiography (*right*). Precipitated proteins were stained with Coomassie Brilliant Blue (*CBB*, *left*). *F* and *G*, mapping of MeCP2 poly(ADP-ribosyl)ated domains. GFP, MeCP2-GFP, and GFP-fused MeCP2 mutants were expressed in HEK293-EBNA (*F*) or MEF (*G*) cells. After immunoprecipitation with GFP-Trap, poly(ADP-ribosyl)ation of the precipitated proteins was checked via Western blotting with anti-poly(ADP-ribose) followed by anti-GFP antibodies. *Top panels*, schematics of fluorescently tagged MeCP2 constructs. *G*, GFP; *Y*, YFP. *Numbers* indicate amino acid coordinates. In the case of lanes that were not next to each other on the original blot, *dashed lines* were employed to indicate that they were moved together to facilitate understanding of the data.

Prompted by the poly(ADP-ribosyl)ation of MeCP2 in U2OS cells reported recently (32), we addressed whether endogenous MeCP2 from mouse brain tissue was poly(ADP-ribosyl)ated. For that, we incubated boronic acid beads, specifically enriching ribonucleotides, with lysates of brain tissue. SDS-PAGE followed by Western blotting using anti-MeCP2 antibody showed that MeCP2 was enriched by boronic acid from mouse brain extracts, illustrating that MeCP2 is modified by ribonucleotides in mouse brain tissue (Fig. 1*A*). To more specifically investigate poly(ADP-ribosyl)ation of endogenous MeCP2 from brain tissue, we performed immunoprecipitation assays of brain extracts with either anti-MeCP2 antibody or control rabbit IgG. Immunoblot analysis with anti-poly(ADP-ribose) antibody showed specific poly(ADP-ribosyl)ation of endogenous MeCP2 (Fig. 1*B*). In addition, we observed poly(ADP-ribosyl)ation of endogenous MeCP2 enriched from mouse brain extracts using Tris-NTA-coupled beads, specifically recognizing the naturally occurring His repeat present within the COOH-terminal domain (amino acids 366–372) of MeCP2 (Fig. 1*C*). We further observed poly(ADP-ribosyl)ation of ectopically expressed MeCP2-GFP but not of GFP alone (Fig. 1*D*).

To determine whether PARP-1 is responsible for MeCP2 poly(ADP-ribosyl)ation, we performed *in vitro* poly(ADP-ribosyl)ation analysis using recombinant MeCP2-GFP in the presence of strep-PARP-1 and [α-^32^P]NAD^+^ (Fig. 1*E*). Although GFP was not modified, PARP-1 specifically poly(ADP-ribosyl)ated MeCP2, which is in agreement with the *in vitro* poly(ADP-ribosyl)ation of MeCP2 published recently (32).

Subsequent mapping identified the domain spanning the interdomain (ID) and TRD to be strongly poly(ADP-ribosyl)ated *in vivo* (Fig. 1*F*). Interestingly, the NH_2_ terminus plus MBD and the COOH terminus showed almost no poly(ADP-ribosyl)ation. We could further narrow down the modified domain to the ID (amino acids 163–206, poly(ADP-ribosyl)ated domain 1) and, to a lesser extent, to amino acids 244–275 (poly(ADP-ribosyl)ated domain 2) (Fig. 1*F*).

Next we tested deletion constructs lacking the poly(ADP-ribosyl)ated regions (Fig. 1*G*). Although deletion of poly(ADP-ribosyl)ated domain 2 (MeCP2G.17, deletion of amino acids 244–275) resulted in slightly less poly(ADP-ribosyl)ation than deletion of the full-length domain, the construct lacking poly(ADP-ribosyl)ated domain 1 (MeCP2G.16, deletion of amino acids 163–206) showed a strong decrease, and the double deletion (MeCP2G.18) had an even stronger effect (Fig. 1*G*).

##### Poly(ADP-ribosyl)ation of MeCP2 Reduces Clustering of Pericentric Heterochromatin

**FIGURE 2.**
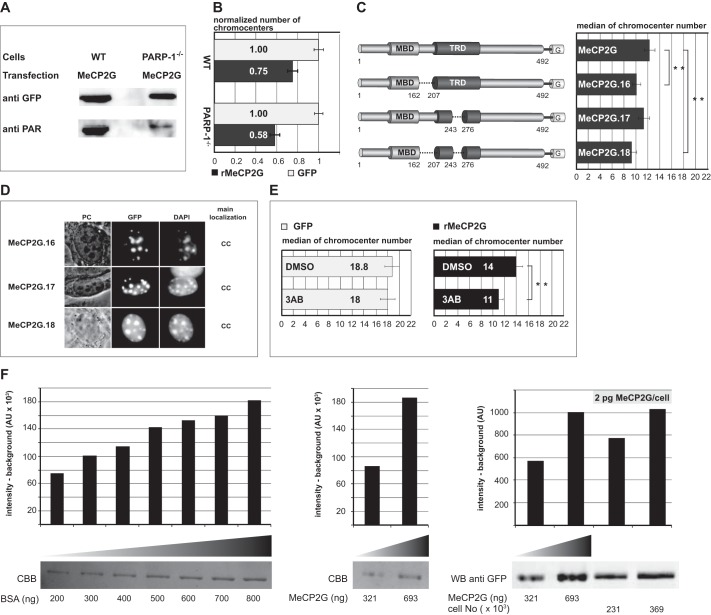
**Poly(ADP-ribosyl)ation counteracts clustering of pericentric heterochromatin.** *A*, MeCP2 exhibits decreased poly(ADP-ribosyl)ation in PARP-1^−/−^ MEF cells compared with WT cells. GFP-fused MeCP2 was expressed in WT and in PARP-1^−/−^ MEF cells. After immunoprecipitation using GFP-Trap, poly(ADP-ribosyl)ation of precipitated proteins was checked via Western blotting with anti-poly(ADP-ribose) (*anti-PAR*) followed by anti-GFP antibodies. *B*, PARP-1^−/−^ MEF cells exhibit enhanced MeCP2-induced chromocenter aggregation in comparison with WT cells. WT or PARP-1^−/−^ MEF cells were transfected with an expression vector coding for GFP or MeCP2-GFP. Z stacks of images were recorded from nuclei with similar high expression levels of the protein using constant image acquisition parameters. Experiments were repeated twice with at least 30 cells/construct each time and are shown normalized to control GFP-expressing cells. *C*, Pmi28 mouse myoblasts were seeded on coverslips and transfected with an expression vector coding for GFP-fused MeCP2G or GFP-fused MeCP2 deletions lacking the poly(ADP-ribosyl)ated domains. Using constant image acquisition parameters, Z stacks of images were recorded of nuclei with similarly high expression levels of the GFP-tagged protein. Graphs show median numbers of chromocenters of cells expressing the indicated proteins. *Error bars* represent 95% confidence interval. Experiments were repeated twice with at least 30 cells/construct analyzed each time. **, *p* < 0.001. *G*, GFP. *D*, overview of the subcellular localization of MeCP2 deletions. Pmi28 myoblast cells were transfected with plasmids coding for MeCP2 deletions fused to GFP as indicated. After fixation, DNA was counterstained with DAPI to highlight chromocenters (*CC*). *PC*, phase contrast. *Scale bar* = 5 μm. *E*, cells were transfected with vectors as indicated and treated with the PARP inhibitor 3AB (10 mm) or dimethyl sulfoxide (*DMSO*) control for about 15 h. Graphs and statistics as in *C. F*, estimation of the amount of GFP-tagged MeCP2 in transfected mouse myoblast cells. Mouse myoblast cells were transfected with plasmids coding for GFP-tagged wild type MeCP2 (*MeCP2G*). GFP-positive cells were isolated and counted using flow cytometry. In parallel, recombinant MeCP2G was purified (using GFP-Trap beads), and its concentration was determined from the mean intensity per ng estimated with a BSA calibration standard using Coomassie Brilliant Blue (*CBB*) staining (*left* and *center panels*). Defined amounts of purified recombinant MeCP2G and MeCP2G expressed in defined numbers of sorted cells were analyzed by SDS-PAGE and quantitative Western blotting with an anti-GFP antibody using fluorescence imaging (*right panel*). The graphs depict the signal intensity (above background) assessed by ImageJ quantitation and presented as arbitrary units (*AU*). All signal intensities were within linear detection range.

Given that MeCP2 is modified by PARP-1 *in vitro* (Fig. 1 and Ref. 32), we next tested whether the absence of PARP-1 in PARP-1^−/−^ mouse fibroblasts might result in a lack of modification with functional consequence on the ability of MeCP2 to reorganize heterochromatin. For that, we compared the numbers of heterochromatic centers in PARP-1^−/−^ mouse fibroblasts expressing either MeCP2-GFP or GFP alone with wild-type cells (Fig. 2*B*). Interestingly, we could measure enhanced aggregation of chromocenters in MeCP2-GFP-expressing PARP-1^−/−^ mouse embryonic fibroblasts, concomitant with reduced poly(ADP-ribosyl)ation levels (Fig. 2, *A* and *B*). We next compared the median numbers of chromocenters in mouse myoblast cells expressing either MeCP2-GFP or one of the deletion constructs lacking the poly(ADP-ribosyl)ated regions (Fig. 2*C*). These adult stem cells express very low to undetectable levels of endogenous MeCP2 (6). First, to rule out that the level of GFP-tagged MeCP2 obtained through ectopic expression in myoblast cells is above the physiological level of MeCP2 in brain neurons, we performed quantitative Western blotting in combination with fluorescence imaging. Using recombinant GFP-tagged MeCP2 as a direct calibration standard for the Western blotting analysis, we could determine the average amount of GFP-tagged MeCP2 in mouse myoblasts to vary between 1.3–2 pg/cell (Fig. 2*F*). These amounts are in the range of endogenous physiological MeCP2 levels per mouse neuronal cell nucleus (33). The numbers of chromocenters in cells expressing the poly(ADP-ribosyl)ated domain 1 deletion (MeCP2G.16) or the double deletion (MeCP2G.18) were reduced significantly compared with cells expressing wild-type MeCP2-GFP (Fig. 2*C*). However, the deletion of poly(ADP-ribosyl)ated domain 2 (MeCP2G.17) had a milder effect. These results correlate well with the poly(ADP-ribosyl)ation levels of the respective constructs (Fig. 1*G*). We could exclude major conformational changes caused by these deletions because all mutant proteins localized at chromocenters as the wild-type protein (Fig. 2*D*).

To further validate that the increase of chromocenter clustering was on the basis of reduced poly(ADP-ribosyl)ation levels and not simply because of deletion of amino acids within MeCP2, we treated cells with the PARP inhibitor 3-amino-benzamide (3AB). Because the chromocenter numbers of GFP-expressing cells treated with 3AB were comparable with dimethyl sulfoxide-treated cells, we concluded that the inhibitors themselves did not have a significant effect on chromocenter aggregation (Fig. 2*E*, *left*). In stark contrast, MeCP2-GFP-expressing cells incubated with the PARP inhibitor exhibited significantly increased clustering of pericentric heterochromatin relative to the dimethyl sulfoxide control (Fig. 2*E*, *right*).

##### MeCP2 Binding to Pericentric Heterochromatin Is Elevated in PARP-1^−/−^ compared with Wild-type Cells

Because the chromatin aggregation potential of MeCP2 was increased in PARP-1^−/−^ mouse fibroblasts in comparison with the wild-type fibroblasts (Fig. 2*B*), we further tested whether the absence of PARP-1 and its mediated posttranslational modification in those cells might have a functional effect on the ability of MeCP2 to bind heterochromatin.

**FIGURE 3.**
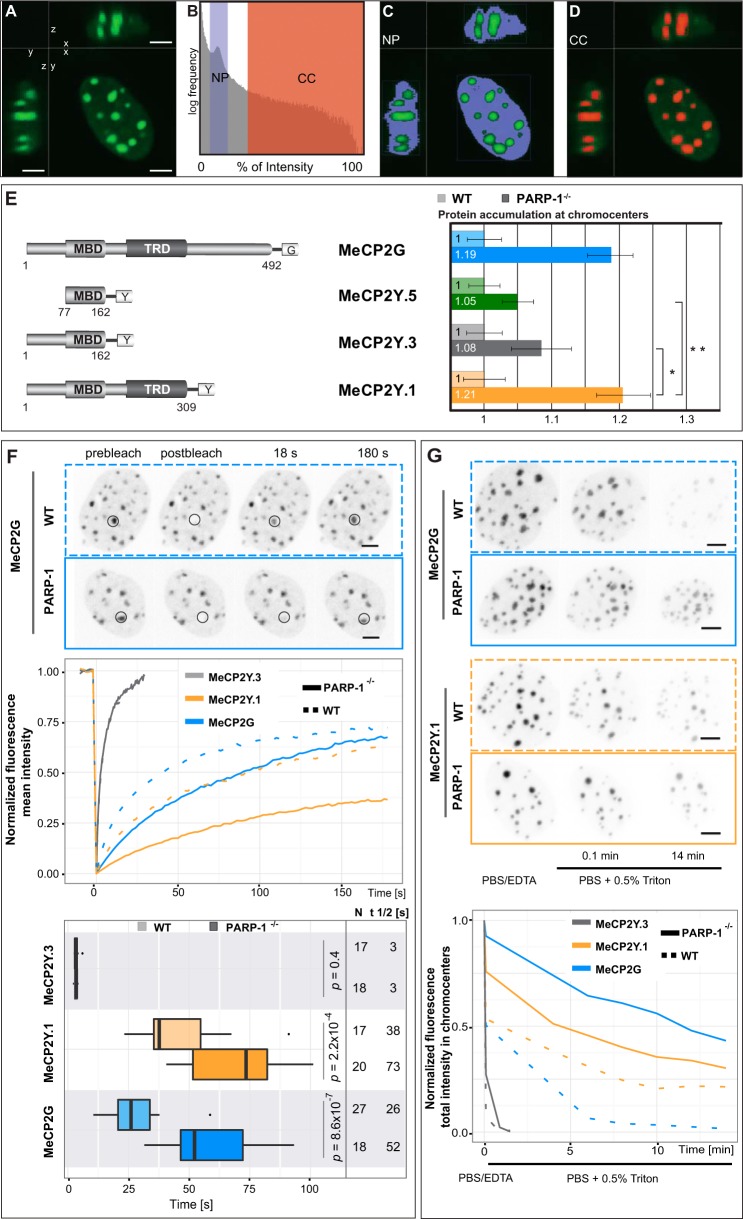
**The chromatin binding ability of MeCP2 is elevated in PARP-1^−/−^ cells.** *A–D*, image analysis protocol for quantification of chromocenter accumulation. All steps necessary for analysis were performed using Volocity 5.5 built-in functions such as image processing, object segmentation, and measurements. Z stacks of cells transfected with a GFP fusion protein were cropped to obtain single nuclei per image. Thresholds for segmentation were set individually for each nucleus. The chromocenter and nucleoplasm were segmented utilizing the built-in thresholding function, which uses the percentage of the overall image intensity. *A*, nucleus of an MeCP2-GFP-expressing cell before segmentation. *Bottom right panel*, the nucleus in *x-y* axis view. *Top right* and *bottom left panels*, the nucleus in *x-z* and *y-z* axis views, respectively. *Scale bars* = 5 μm. *B*, histogram of the frequency of fluorescence intensities plotted on a logarithmic scale. The thresholds for chromocenter (*CC*) and nucleoplasm (*NP*) segmentation are displayed in *red* and *blue*, respectively. Care was taken to exclude chromocenters from nucleoplasm segmentation and vice versa. *C* and *D*, visualization of chromocenter (*red*) and nucleoplasm (*blue*) segmentation according to the thresholds displayed in *B. E*, WT or PARP-1^−/−^ MEF cells were transfected with expression vectors coding GFP- or YFP-tagged MeCP2 constructs as indicated. Z stacks of images were taken from cells expressing comparable levels of the GFP-fused construct. Experiments were repeated at least twice with as many as 30 cells analyzed per construct each time. The graphs show the accumulation of the MeCP2 constructs at heterochromatin in PARP-1^−/−^ cells normalized to wild-type mouse fibroblasts. *, *p* < 0.05; **, *p* < 0.001. *Error bars* represent 95% confidence interval. *F*, FRAP analysis of full-length GFP-tagged MeCP2 and mutant proteins. *Top panel*, an example of MeCP2G protein recovery after photobleaching. *Circles* indicate the bleached region. *Scale bars* = 5 μm. *Center panel*, fluorescence recovery curve for each construct. The experiment was repeated twice with 7–15 cells used each time for analysis. Results were averaged, and the mean value was plotted. *Bottom panel*, the half-time of each construct and *p* value for WT and PARP-1^−/−^ cell line are calculated using a *t* test. The cell number and median are indicated beside the box plot. *G*, *in situ* extraction of full-length GFP-tagged MeCP2 and mutant proteins. *Top panel*, representative images of MeCP2G and MeCP2Y.1 extraction after 0.5% Triton X-100 treatment. *Scale bars* = 5 μm. *Bottom panel*, normalized fluorescence intensity before and after treatment. For each construct, the normalized intensity was averaged, and the mean value was plotted. The experiment was repeated twice, and each time 5–10 cells were used for analysis.

Therefore, we transfected wild-type and PARP-1^−/−^ mouse fibroblasts with a plasmid coding for GFP-labeled MeCP2 and quantified MeCP2 binding to heterochromatin *in vivo* (Fig. 3, *A–D*). We found that the heterochromatin accumulation ability of MeCP2-GFP was increased significantly in PARP-1^−/−^ compared with wild-type cells (Fig. 3*E*). This is not the result of differences in DNA methylation *per se* at major satellite repeats in PARP-1^−/−^ *versus* wild-type cells because it has been reported recently that cytosine methylation is unchanged (34).

To test whether the poly(ADP-ribosyl)ated domains were needed, we selected MeCP2 deletions, including or excluding the modifiable ID-TRD. To allow scoring of chromatin binding *in vivo*, we further needed to include the MBD (6, 14). Comparing all of these deletions in PARP-1^−/−^ cells, it became clear that the ID-TRD was responsible for a significant enhancement in chromatin binding (Fig. 3*E*). These data indicate that the observed effect is not due to methylcytosine binding but that it requires PARP-1 and the ID-TRD of MeCP2.

To further probe MeCP2 heterochromatin binding kinetics, we performed fluorescence recovery after photobleaching (FRAP) analyses of GFP-tagged MeCP2 as well as MeCP2 deletions with or without the modifiable ID-TRD. The results showed that the ability of MeCP2-GFP to accumulate to pericentromeric heterochromatin was accelerated in PARP-1^−/−^ compared with wild-type cells, as evident by the slower recovery after photobleaching in PARP-1^−/−^ cells (Fig. 3*F*). Strikingly, the MeCP2 deletion construct, including the ID-TRD, also showed a prominent enhancement in chromatin binding in PARP-1^−/−^ compared with wild-type cells, whereas the deletion construct terminating after the MBD domain did not exhibit any altered chromatin accumulation and displayed a very fast exchange at heterochromatin (Fig. 3*F*).

Finally, we performed *in situ* extraction experiments of PARP-1^−/−^ and wild-type cells expressing the same full-length MeCP2 and deletion mutants. Both GFP-tagged full-length MeCP2 and the deletion containing the ID-TRD domain (MeCP2Y.1) were extracted after several minutes of Triton X-100 incubation. Importantly, they were extracted faster from wild-type cells and were still well detectable at chromocenters after 14 min of detergent treatment in PARP-1^−/−^ cells (Fig. 3*G*). In stark contrast, the mutant truncated after the MBD was very fast and extracted equally from both wild-type and PARP-1^−/−^ cells.

Together, the results obtained from these three independent methods addressing chromatin binding clearly indicate that MeCP2 chromatin accumulation depends on PARP-1 and the ID-TRD of MeCP2 and that the ID-TRD alone is sufficient to restore MeCP2 heterochromatin binding ability *in vivo* to a comparable degree as the full-length protein.

## Discussion

**FIGURE 4.**
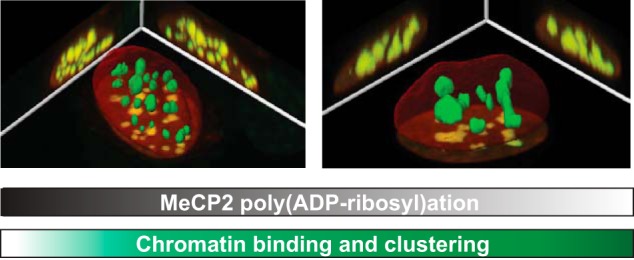
**Summary of factors influencing MeCP2-mediated heterochromatin binding and clustering.** A higher level of MeCP2 and/or a decrease of MeCP2 poly(ADP-ribosyl)ation causes increased heterochromatin binding and clustering (hyperclustering).

In summary, we showed poly(ADP-ribosyl)ation of MeCP2 in mouse brain tissue (Fig. 1). In addition, we found that MeCP2-induced pericentric heterochromatin clustering is increased in the absence of PARP-1 (Fig. 2) and could show that the chromocenter binding ability of MeCP2 is elevated in PARP-1^−/−^ in comparison with wild-type cells (Fig. 3). Because a reduced poly(ADP-ribosyl)ation level of MeCP2 led to a significant but not too strong increase in the chromatin aggregation ability of MeCP2, we suggest a modulatory role of poly(ADP-ribosyl)ation in MeCP2-mediated chromocenter aggregation (Fig. 4).

We propose that varying degrees of poly(ADP-ribosyl)ation within MeCP2 establish different anionic phosphate-containing islands within the highly cationic MeCP2 protein. This should lower MeCP2 affinity to negatively charged DNA and/or chromatin proteins (3–5). Because nucleosome-MeCP2-nucleosome or DNA-MeCP2-DNA interactions have been proposed as a mechanism for MeCP2-induced chromatin compaction *in vitro* (11), we demonstrate here that poly(ADP-ribosyl)ation could interfere with these interactions and uncluster chromatin *in vivo*. Although the MBD domain is necessary and sufficient for chromatin clustering *in vivo* (6), different lines of evidence point to an additive function of the ID-TRD in this context. On one hand, fluorescence recovery after photobleaching studies demonstrated that, in addition to the MBD, the ID and TRD also strengthen MeCP2 chromatin binding *in vivo* (Fig. 3 and Refs. 12, 13). On the other hand, *in vitro* biochemical analyses showed that the ID and TRD contribute to DNA and nucleosomal interactions (35). The fact that poly(ADP-ribosyl)ation targets both of these domains suggests a regulatory role of this modification in the MeCP2 chromatin remodeling function. Our results showing an increase of chromatin binding in PARP-1^−/−^ compared with wild-type cells reinforce this proposal.

Most nonsense and frameshift mutations reported in Rett syndrome truncate MeCP2 after the MBD, and, in particular, the nonsense mutations R168X and R255X are among the most frequent mutations in Rett syndrome patients. This suggests that aberrant MeCP2 poly(ADP-ribosyl)ation could additionally contribute to protein dysfunction in Rett syndrome, as has been proposed recently for phosphorylation of MeCP2 (28–30).

Our data reveal a complex interplay between MeCP2 domains, their regulation by poly(ADP-ribosyl)ation, and the functional consequences for MeCP2-mediated, higher-order chromatin organization. We propose that residues within the MBD domain of MeCP2 and poly(ADP-ribosyl)ation within the ID and TRD work in concert to mediate and regulate MeCP2 function in modeling chromatin architecture. Because we have shown recently that the ID-TRD mediates homo-interactions of MeCP2 (19), it is tempting to speculate that poly(ADP-ribosyl)ation within this domain negatively regulates the ability of MeCP2 to cross-link chromatin fibers *in vivo*.

## Author Contributions

A. B. conducted most of the experiments, analyzed the results, and wrote most of the paper. P. Z. performed and analyzed the FRAP and *in situ* extraction experiments. L. A. conducted and, with the help of B. B. and D. E., analyzed the chromocenter binding microscopy experiments. D. M. and H. L. provided the sorted cell material for MeCP2 quantification. M. Hofstaetter contributed baculovirus constructs and insect cell expression. G. B. and M. Hottiger provided the boronic acid data. V. S. provided materials and protocols. M. C. C. designed the project and wrote the paper with A. B.

## Footnotes

4 The abbreviations used are:

MBD: methyl CpG binding domain
TRD: transcriptional repression domain
PARP: poly(ADP-ribose) polymerase
MEF: mouse embryonic fibroblast
NA: numerical aperture
NTA: nitrilotriacetic acid
ID: interdomain
3AB: 3-amino-benzamide
FRAP: fluorescence recovery after photobleaching.
